# The captopril challenge test for diagnosing primary Aldosteronism in a Chinese population

**DOI:** 10.1186/s12902-019-0390-3

**Published:** 2019-06-24

**Authors:** Ke-ying Zhu, Yan Zhang, Wen-jing Zhang, Hong-yun Li, Wen-huan Feng, Da-long Zhu, Ping Li

**Affiliations:** 1 Department of Endocrinology, Drum Tower Hospital Affiliated to Nanjing University Medical School, Nanjing, 210008 People’s Republic of China; 2 Department of Endocrinology, Drum Tower Hospital Affiliated to Nanjing Medical University School, Nanjing, 210008 People’s Republic of China

**Keywords:** Primary aldosteronism, Diagnosis, Captopril challenge test

## Abstract

**Background:**

The Captopril challenge test (CCT) is an easy-conduct confirmatory test for diagnosing primary aldosteronism (PA). Guidelines show that plasma aldosterone is normally suppressed by captopril (> 30%) in primary hypertension (PH) and in healthy people. It is unclear whether this standard is applicable in Chinese subjects. The aim of the present study was to investigate the post-CCT efficacy of plasma aldosterone concentration (PAC) suppression and determine the post-CCT aldosterone renin activity ratio (ARR) and PAC for PA diagnosis.

**Methods:**

We recruited 110 consecutive patients with PA, 163 with primary hypertension (PH), and 40 healthy volunteers (NC). The CCT was conducted in all patients. Total sodium intake was estimated from 24-h urinary excretions. ROC curves were used to analyze the efficiency of different CCT diagnostic criteria for diagnosing PA.

**Results:**

In NC and PH patients, PRA was increased and PAC was decreased post-CCT (*P* < 0.05). The mean degree of PAC decline after CCT was approximately 9.3%, and only 11.7% of the patients with PH showed a greater than 30% suppression of PAC after CCT. In patients with PA, the post-CCT change in PRA and PRC was slight. The post-CCT degree of PAC decline was unrelated to dietary salt intake. The areas under the ROC for the post-CCT ARR, PAC and PAC suppression % were 0.994, 0.754 and 0.606, respectively. The optimal post-CCT cutoff value for ARR for diagnosing PA was 20, which yielded a sensitivity and specificity of 94.0 and 99.4%, respectively.

**Conclusions:**

The PAC suppression percentage after CCT recommended by current clinical guidelines is not applicable when diagnosing Chinese subjects with PA. Compared to post-CCT PAC, post-CCT ARR was a better approach, having an optimal cutoff of 20 when interpreting the results of the CCT in Chinese patients. We found no relationship between high salt intake and low responses of the renin-angiotensin system (RAS) to the CCT.

## Background

Primary aldosteronism (PA) is the most common cause of secondary hypertension and affects approximately 10% of patients with essential hypertension [1]. In resistant hypertensive cohorts, the prevalence of PA is approximately 20% [2]. In addition to its high prevalence, patients with PA have higher cardiovascular morbidity and mortality than are reported in patients with essential hypertension and the same degree of blood pressure (BP) elevation [3, 4]. Furthermore, specific treatments are available that can cure hypertension and ameliorate its complications [5]. Therefore, early and accurate diagnosis is imperative.

A previous study demonstrated that in China, the overall prevalence rate of PA in adults with resistant hypertension was 7.1% [6], which is not the highest among those previously reported in other ethnic populations outside China [7, 8]. However, China has a large general population with a prevalence of hypertension of 29.6% [9]. It is challenging to accurately and quickly diagnose PA in such a large population. Hence, convenient diagnostic processes with a high efficiency for identifying PA are currently urgently needed in China.

Confirmation tests are a crucial step in the process of obtaining a functional diagnosis of PA. The Endocrine Society’s guidelines for PA recommend 4 confirmatory tests, including the fludrocortisone suppression test (FST), saline infusion test (SIT), captopril challenge test (CCT), and oral sodium loading. Among these confirmatory tests [1], the CCT is easy-to-conduct and is considered to have the same diagnostic efficiency as the other tests [1]. However, there is no consensus on how the results of the CCT should be interpreted [1, 10, 11]. The Endocrine Society guidelines recommend that a post-CCT suppression percentage < 30% confirms PA, but this threshold has not been verified in studies [1]. However, other groups prefer to use the post-CCT aldosterone renin activity ratio (ARR) or post-CCT plasma aldosterone concentration (PAC) to diagnose PA [12–15]. Diets and genetic susceptibilities are different between Chinese and Western populations [16], and previous studies have suggested that Chinese and Western individuals perform differently on the SIT [17]. Therefore, the best approach to using and cutoff value for the CCT in Chinese patients have not yet been defined.

The present study aimed to investigate the usefulness of, optimal interpretation approach to, and cutoff value for the CCT in diagnosing PA in Chinese patients. Furthermore, the correlation between estimated sodium intake and CCT performance was also assessed.

## Methods

### Subjects

The present study was conducted in the Nanjing Drum Tower Hospital. All subjects provided informed consent. We retrieved data from hypertensive patients who were referred to our unit from January 2011 to December 2016 with a suspicion of PA because of resistant hypertension, spontaneous or diuretic-induced hypokalemia, or the finding of an adrenal mass. Potassium-wasting or -sparing diuretics were discontinued at least 4 weeks prior to testing. Other drugs, including β-blockers, angiotensin-converting enzyme inhibitors and angiotensin receptor blockers, were withdrawn for at least 2 weeks. Patients with severe hypertension were prescribed α-blockers, and long-acting calcium channel blockers were prescribed if necessary. Dietary sodium intake was unrestricted. Patients with hypokalemia were given adequate oral or intravenous potassium supplements to maintain serum potassium levels higher than 3.0 mmol/L before the study was begun.

Data representing normal controls were retrieved from healthy volunteers who participated in the study and had a BP below 140/90 mmHg and no history of hypertension and were not receiving any medication. Finally, we included 110 PA patients, 163 primary hypertension (PH) patients and 40 normal controls (NC).

### Biochemical measurements

Sodium and potassium levels in blood and urine were measured using fully automated instrumentation. Plasma renin activity (PRA) was measured by radioimmunoassay using a commercial kit (Atomic hi-tech Co., LTD, Beijing, China). The intra- and inter-assay coefficients of variation (CVs) for PRA were 10 and 15%, respectively. PAC was measured by radioimmunoassay with commercial kits (Northern Technical and Biological Institute, Beijing, China). The intra-assay and inter-assay CVs for this assay were 5.6 and 6.2%, respectively.

### Diagnostic procedures

Blood samples for PRA and PAC were obtained in all patients between 0800 and 0900 h after overnight fasting and at least 2 h lying in a supine position and then again after 2 h of keeping in an upright position. The CCT was performed in the morning. Patients received 50 mg of captopril orally after they were upright (sitting, standing, or walking) for at least 2 h and seated for 5–15 min. Blood samples were drawn to measure PRA and PA at time 0 and at 2 h after challenge. The patient remained seated during this period.

All hypertensive patients underwent an enhanced adrenal computed tomography (CT) scan with fine cuts (3 mm). Adrenal vein sampling (AVS) procedures were performed between 0800 and 1200 h by one radiologist using the bilateral simultaneous technique without cosyntropin stimulation. According to previous recommendations, successful cannulation was considered a selectivity index (SI) ≥ 2, and lateralization of aldosterone secretion was defined as a lateralization index (LI) ≥ 2.

PH was defined as a systolic and diastolic BP greater than 140/90 mmHg according to the established criteria and excluding secondary hypertension, such as renal parenchymal hypertension, renovascular hypertension, endocrine hypertension, aortic coarctation, sleep apnea syndrome, and drug-induced hypertension.

Identification of PA required all of the following criteria: (1) ARR ≥ 25 and PAC > 12 ng/dL in addition to (2) at least one of the following additional endocrine alterations: a. upright PRA < 1.0 ng/ml/h, b. ARR post-CCT ≥ 20 or post-CCT PAC suppressed less than 30%.

Identification of aldosterone-producing adenoma (APA) was based on the following criteria: (1) evidence of PA as defined above, (2) lateralization of aldosterone secretion at CT and/or AVS, (3) evidence of adenoma at CT and/or surgery and/or pathology, and (4) demonstration of normokalemia and hypertension cure or improvement or normalization of PAC and PRA at follow-up after adrenalectomy.

Identification of idiopathic hyperaldosteronism (IHA) was based on the following criteria: (1) evidence of PA as defined above, (2) bilateral adrenal lesions on CT scan and/or aldosterone secretion at AVS, (3) demonstration of normokalemia and improvement of hypertension after treatment with mineralocorticoid receptor antagonists.

### Statistical analysis

Normally distributed data are expressed as the means ± standard deviation, and abnormally distributed data are expressed as a median (interquartile intervals 25–75%). Values between groups were compared by one-way analysis of variance for parametric data and the Kruskall-Wallis test for nonparametric data. The diagnostic accuracy of post-CCT PAC suppression (as a percentage), PAC and ARR for identifying PA was assessed with a receiver-operated characteristic (ROC) curve and the area under the ROC curve (AUC). The best cutoff and both sensitivity and specificity are provided. A value of *P* < 0.05 was considered to be statistically significant. Most statistical analyses were performed using the software package SPSS 21.0 for Microsoft Windows (SPSS Inc.). The ROC curve comparison was performed with MedCalc software 15.2.2 (MedCalc Software).

## Results

### Clinical characteristics of patients with PA

We recruited 110 consecutive patients with PA. Of these patients, 82 had an APA, and 28 had IHA. The clinical characteristics of each group are summarized in Table 1. There was no significant difference among the groups in age, sex and BMI. Systolic blood pressure (SBP) and diastolic blood pressure (DBP) were higher in the PA and PH groups than in the NC group (*P* < 0.01). BP was higher in the APA group than in the PH group (*P* < 0.01). Compared to the NC and PH groups, patients in the PA group had lower serum potassium and higher serum sodium and urine potassium levels (*P* < 0.01). PAC was markedly higher and PRA was significantly lower in the PA group than in the PH and NC groups (*P* < 0.01). The ARR was consequently much higher in the PA group than in the PH and NC groups (*P* < 0.05). The prevalence of hypokalemia was higher in the APA group than in the IHA group (92.7% vs 75%). Compared to patients with IHA, those with APA had higher PAC (*P* < 0.01). No other significant differences were detected between the APA and IHA groups.

**Table 1:** Clinical characteristics of the included normal controls and patients

	NC (*n* = 40)	PH (*n* = 163)	IHA (*n* = 28)	APA (*n* = 82)
Age (y)	49.0 ± 11.5	48.6 ± 15.4	51.5 ± 10.9	46.0 ± 12.9
Sex (M/F)	19 / 21	92 /71	18 / 10	37 / 45
BMI (kg/m^2^)	25.0 ± 3.6	25.6 ± 4.0	25.2 ± 2.4	25.5 ± 8.9
SBP (mmHg)	124.2 ± 9.3	145.4 ± 19.6^A^	144.5 ± 13.4^A^	152.0 ± 20.8^AB^
DBP (mmHg)	75.1 ± 9.5	89.0 ± 14.4^A^	90.3 ± 11.9^A^	94.7 ± 15.3^AB^
Prevalence of hypokalemia (%)	–	–	75.0	92.7^c^
Serum potassium (mmol/L)	4.0 ± 0.29	4.0 ± 0.25	3.3 ± 0.45^AB^	3.2 ± 0.53^AB^
Serum sodium (mmol/L)	141.1 ± 1.9	141.0 ± 1.8	143.2 ± 2.3^AB^	143.5 ± 3.7^AB^
Urine potassium (mmol/24 h)	36.7 ± 12.9	38.9 ± 15.6	57.5 ± 27.0^AB^	58.2 ± 26.8^AB^
Supine PRA (ng/ml.h)	1.0 (0.55~1.9)	1.4 (0.65~2.4)	0.11 (0.03~0.26)^AB^	0.14 (0.025~0.33)^AB^
Supine PAC (ng/dl)	11.2 ± 4.7	12.7 ± 3.8	15.0 ± 6.1^Ab^	17.8 ± 5.5^ABC^
ARR	10.6 (5.4~17.1)	8.9 (5.4~17.5)	84.6 (64.6~556.2)^AB^	117.5 (49.4~695.8)^AB^

Data are expressed as the mean ± SD or median (25th–75th percentiles). *NC* normal controls, *PH* primary hypertension, *IHA* idiopathic hyperaldosteronism, *APA* aldosterone-producing adenoma, *BMI* body mass index, *SBP* systolic blood pressure, *DBP* diastolic blood pressure, *PRA* plasma renin activity, *PAC* plasma aldosterone concentration, *ARR* aldosterone to renin ratio. ^A^*P* < 0.01, ^a^*P* < 0.05 vs. NC; ^B^*P* < 0.01, ^b^*P* < 0.05 vs. PH; ^C^*P* < 0.01, ^c^*P* < 0.05 vs. IHA

### Captopril challenge test in the normal control and patient groups

In the NC and PH groups, PRA was higher and PAC was lower after CCT (P < 0.05). As a result, ARR was lower post-CCT than pre-CCT (*P* < 0.01). However, the mean post-CCT suppression percentage of PAC was only approximately 9.3%, and only 11.7% of the patients with PH showed a post-CCT decrease in PAC greater than 30%. In PA patients, the CCT increased PRA and lowered PAC slightly. After CCT, PAC remained higher and PRA was lower in the PA group than in the PH and NC groups (*P* < 0.01). As a result, ARR remained higher post-CCT higher in the APA and IHA groups than in the PH and NC groups (*P* < 0.01). The post-CCT PAC was still higher in the APA group than in the IHA group (*P* < 0.05). No other differences were detected in the indexes between the APA and IHA groups after CCT (Table 2).

**Table 2:** Results of the captopril challenge test (CCT) in normal controls and patients

	Pre-CCT			Post-CCT			PAC suppression percentage (%)
	PRA (ng/ml.h)	PAC (ng/dl)	ARR	PRA (ng/ml.h)	PAC (ng/dl)	ARR	
NC (n = 40)	1.4 (0.91~4.2)	13.2 ± 4.7	6.9 (3.9~14.4)	3.0 (1.6~5.3) *	12.1 ± 4.4†	3.9 (1.8~7.3) †	10.2 (− 2.6~20.8)
PH (n = 163)	2.3 (1.2~3.6)	13.6 ± 3.9	6.1 (3.5~10.2)	3.3 (1.8~5.7) †	12.2 ± 4.1†	3.4 (2.2~7.0) †	10.6 (− 1.4~21.5)
IHA (n = 28)	0.23 (0.05~0.36)^AB^	15.5 ± 6.4^ab^	60.0 (34.8~169.2)^AB^	0.31 (0.20~0.58)^AB^†	14.9 ± 5.6^aB^	41.1 (27.9~61.2)^AB^	3.4 (−15.1~17.1)
APA (n = 82)	0.21 (0.048~0.35)^AB^	18.2 ± 4.9^ABC^	80.0 (47.7~263.9)^AB^	0.30 (0.080~0.53)^AB^†	17.4 ± 5.0^ABc^†	54.7 (35.2~196.9)^AB^	3.8 (−5.3~11.7)^b^

Data are expressed as the mean ± SD or median (25th–75th percentiles). ^A^*P* < 0.01, ^a^*P* < 0.05 vs. NC; ^B^*P* < 0.01, ^b^*P* < 0.05 vs. PH; ^C^*P* < 0.01, ^c^*P* < 0.05 vs. IHA; *P < 0.05, †*P* < 0.01 vs pre-CCT in each group

### Effects of sodium intake on CCT performance

We divided our subjects according to daily urinary sodium excretion to investigate the effect of sodium intake on CCT performance. Patients were divided into low and high sodium intake groups according to median daily urinary sodium. There were no significant differences between the PA and PH patient groups and the NC group in either pre- or post-CCT PAC, PRA and ARR values. Neither the PAC suppression percentage nor the PRA incretion percentage differed among the PA, PH and NC groups (Table 3).

**Table 3:** Results of the CCT according to median Na ^+^ Intake

	APA and IHA		PH and NC	
	Below median (*n* = 54)	Above median (*n* = 53)	Below median (*n* = 102)	Above median (*n* = 101)
Urine sodium (mmol/24 h)	106.3 ± 30.0	247.6 ± 76.7^A^	109.4 ± 27.7	205.5 ± 41.5^A^
Pre-PAC (ng/dl)	17.7 ± 5.9	17.4 ± 5.0	13.6 ± 4.0	13.4 ± 4.2
Pre-PRA (ng/ml.h)	0.20 (0.048~0.42)	0.23 (0.065~0.35)	2.2 (0.96~3.7)	2.3 (1.2~3.7)
Pre-ARR	63.1 (45.1~300.4)	81.5 (48.0~165.2)	6.8 (3.7~13.1)	5.9 (3.5~10.1)
Post-PAC (ng/dl)	17.2 ± 5.7	16.4 ± 5.0	12.1 ± 4.0	12.2 ± 4.3
Post-PRA (ng/ml.h)	0.28 (0.050~0.62)	0.31 (0.18~0.49)	3.4 (1.6~6.1)	3.0 (1.8~5.0)
Post-ARR	50.1 (28.5~223.3)	52.0 (32.1~89.9)	3.1 (1.9~7.2)	3.7 (2.2~6.9)
PAC suppression percentage (%)	3.7 (−8.1~11.8)	3.4 (−6.1~13.3)	12.9 (−0.73~20.3)	7.9 (−3.0~21.9)
PRA incretion percentage (%)	25.1 (−21.3~102.3)	50.5 (0.0~129.0)	55.0 (9.3~158.0)	53.0 (10.1~112.1)

^A^*P* < 0.01 vs. below median

### Diagnostic accuracy of the CCT

**Fig. 1:**
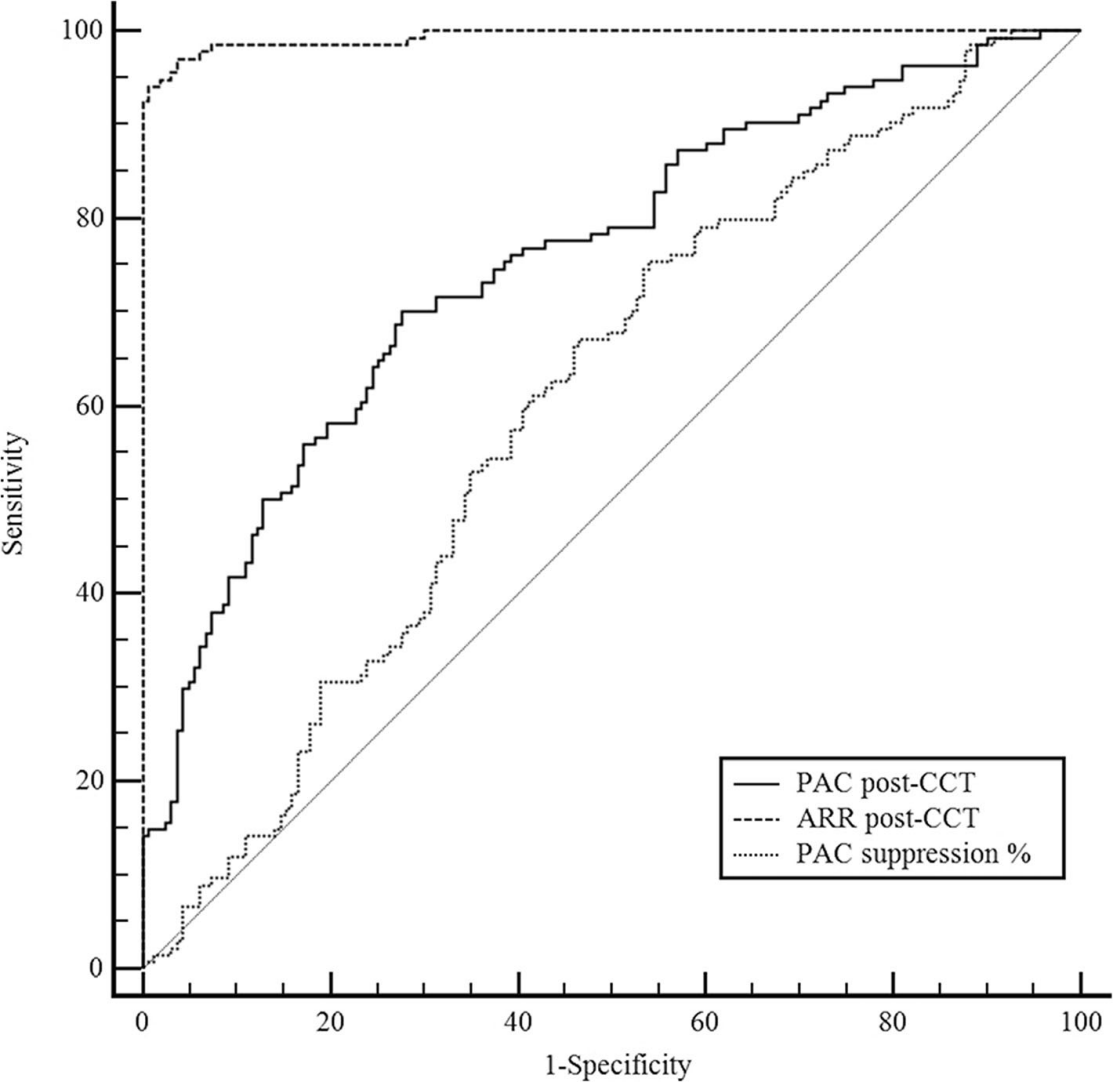
ROC curve analysis of 3 diagnostic criteria in the CCT used for PA diagnosis. PAC suppression %: plasma aldosterone concentration suppression percentage in the captopril challenge test, PAC post-CCT: plasma aldosterone concentration after the captopril challenge test, ARR post-CCT: aldosterone–renin ratio after the captopril challenge test

According to the Endocrine Society Clinical Practice Guidelines, a post-CCT PAC suppression percentage less than 30% is recommended to confirm PA.^1^ Other association guidelines recommend the use of post-CCT ARR or PAC for diagnosing PA [10, 11]. Using ROC curves, we investigated the efficiency of different diagnostic criteria in the CCT for diagnosing PA. The AUC of the PAC suppression percentage was only 0.606, which suggests that the post-CCT PAC suppression percentage is of no value for diagnosing PA in our population. However, when the post-CCT ARR or PAC was used, the AUC of the CCT increased to 0.994 (95% CI, 0.987–1.000, *P* < 0.001) and 0.754 (95% CI, 0.699–0.809, *P* < 0.001), respectively. The AUC of the post-CCT ARR was larger than that of the post-CCT PAC (*P* < 0.01) (Fig. 1). The optimal cutoff value for the post-CCT ARR for diagnosing PA was 20, with a sensitivity and specificity of 94.0% (95% CI, 88.58–97.39%) and 99.4% (95% CI, 96.63–99.98%), respectively. The optimal cutoff for the post-CCT PAC was set at 13 ng/dL, which yielded a sensitivity and specificity of 70.1% (95% CI, 61.64–77.74%) and 72.4% (95% CI, 64.86–79.1%), respectively. These values were lower than those for the post-CCT ARR (*P* < 0.001).

## Discussion

Four testing procedures (oral sodium loading, SIT, FST, and CCT) are recommended by the Endocrine Society’s 2008 and 2016 clinical guidelines for PA confirmation diagnosis [1, 18]. Until recently, insufficient direct evidence was available for recommending any one of these tests over all others. The choice of confirmatory test for PA diagnosis is commonly determined after considering cost, patient compliance, routine laboratory tests, and local expertise, as suggested by the guidelines [1]. Because it is safe, cost-effective, and feasible to perform, the CCT is widely used for PA diagnosis in China. The results of the current study reveal that the post-CCT degree of PAC suppression below 30% recommended by the Endocrine Society is ineffective in diagnosing PA in Chinese patients with hypertension. Compared to post-CCT PAC, post-CCT ARR is a more reliable metric for diagnosing PA in Chinese populations.

The diagnostic efficiency of the CCT varies substantially among previous studies [12–15, 19–21]. This may be because of differences in the doses of captopril (25 or 50 mg), in the time point for blood draws (i.e., 60, 90, or 120 min after captopril administration), in the method used to interpret the results (i.e., PAC suppression percentage, post-CCT PAC, or post-CCT ARR), and in the criteria used to diagnose PA. The Endocrine Society’s clinical guidelines concluded that CCT has the same diagnostic efficiency as other confirmation tests and suggested using the PAC suppression percentage as a clinical diagnostic approach. PA is diagnosed by CCT when the PAC is not suppressed by captopril (< 30%) [1, 18]. The results of the present study showed, after CCT, the PAC decreased by 3.8% in the APA group, 3.4% in the IHA group, 10.6% in the PH group and 10.2% in the NC group. These differences in the percentage suppression of the PAC among the four groups were not significant. These findings indicate that using a suppression percentage of 30% as the approach cutoff may cause many hypertensive patients to be misdiagnosed. Two recently published studies support our results. Both found that the percentage suppression in the PAC after CCT was low in Chinese patients [22, 23]. Given that our study included patients recruited from areas of China different from those in the other two studies, our data provide additional new evidence supporting the conclusion that using a post-CCT PAC suppression percentage > 30% as a criteria is not appropriate for diagnosing PA in Chinese patients.

In addition to our study, other data collected in China have demonstrated that PAC suppression in the CCT or SIT is lower in Chinese than in Western patients [17, 22, 23]. The mechanism underlying this variation between Chinese and Western populations has not been clarified. A high salt intake has been postulated to inhibit renin-angiotensin system activity and to thereby alter the results of confirmation tests used to diagnose PA [24–26]. Rossi et al. showed that the accuracy of the CCT for PA diagnosis was affected by sodium intake. The optimal aldosterone cutoff value for PA diagnosis was elevated in individuals with a higher median daily sodium urinary excretion [15]. The Chinese diet is high in sodium. Our and other studies performed in China have demonstrated that 24-h urine sodium excretion (widely used to estimate daily sodium consumption) is higher in China than in Western countries [16, 27]. It is logical to speculate that a long-term high salt diet might be one of the factors that contributes to the lower response to the CCT observed in Chinese subjects. However, when we split our patients according to the median daily urinary sodium, we found that plasma aldosterone and PRA values were not different between low- and high-sodium intake patients. Li et al. confirmed the same phenomenon in their study [17]. They found no evidence that the lower response of the renin-angiotensin system (RAS) to the SIT was related to high-salt intake [17]. Some possible explanations for the fact that sodium intake had no effect on RAS activity in our and other studies include the following: 1) a single 24-h urine sodium excretion measurement will not accurately reflect long-term sodium intake levels and may therefore bias the results; 2) Our and Li’s studies were all conducted in hospitalized patients. It is difficult to identify differences among patients consuming almost the same relative high-sodium diet. Further studies with a crossover and prospective design are needed to evaluate the effects of low- and high-sodium intake on CCT or SIT performance. In addition to sodium intake, other factors, such as genetic susceptibility, that might be related to the observed discrepancies in performance on PA confirmation tests between Chinese and Western populations should also be considered.

The principle the captopril test is based is the suppression of the serum concentration of aldosterone and an increase in the level of PRA when angiotensin-converting enzyme is inhibited in patients without autonomous aldosterone secretion [1]. Previous studies and two newly published studies performed in China have recommended using the post-CCT PAC to confirm a diagnosis of PA, but they have suggested using various cutoff values [14, 22, 23]. Other groups prefer post-CCT ARR for PA diagnosis [12, 13, 15]. The Japan and Taiwan Endocrine Society recommend the use of post-CCT PAC or post-CCT ARR for PA diagnosis [10, 11]. We compared the performance of these two approaches in our study. The results showed that post-CCT ARR was superior to post-CCT PAC (AUC = 0.994 vs 0.754), inconsistent with the study by Song et al., who found that while post-CCT ARR is useful, post-CCT PAC has a higher sensitivity than does post-CCT ARR in PA diagnosis [22]. Our and Song’s studies both detected only a small amount of post-CCT suppression in PAC but a dramatic increase in post-CCT PRA or PRC in normal and PH patients. Therefore, it is reasonable to suspect that post-CCT ARR is a more sensitive marker than post-CCT PAC for differentiating PA patients from normal or PH patients. The possible reasons for the discrepancy between our results and those of Song et al. may include the following: 1) Song et al. used FST instead of a conclusive PA diagnosis as the reference standard, and this may have biased their data interpretation; and 2) the plasma renin concentration (PRC) was measured instead of PRA in Song’s study, and their ARR was different from ours.

The optimal post-CCT ARR cut-off value is variable among studies that support the use of post-captopril ARR as a PA diagnostic tool. The divergence in cutoff values may be associated with the assays and techniques used to measure aldosterone and PRA. Consistent with the guidelines of the Japan Endocrine Society, we found that the best cutoff for the post-CCT ARR was 20, which yielded relatively high sensitivity (94.0%) and specificity (99.4%) in this study. Therefore, we found that the post-CCT ARR is a reliable marker for diagnosing PA in a Chinese population.

## Conclusion

In summary, in this study, we demonstrate that CCT is a reliable functional test for confirming a PA diagnosis. Compared to Western populations, Chinese populations exhibit lower degrees of post-CCT PAC suppression. As a result, the post-CCT PAC decline below 30% recommended as a threshold by the Endocrine Society is ineffective for diagnosing PA in Chinese hypertension subjects. Compared to the post-CCT PAC, the post-CCT ARR is a better approach when used with an optimal cutoff of 20 for interpreting the results of the CCT in Chinese patients. Furthermore, there was no relationship between high salt intake and lower RAS responses in this study. Further study is needed to identify the factors underlying this variation between Chinese and Western populations.

## Data Availability

The datasets analyzed during the current study are available from the corresponding author on reasonable request.

## Abbreviations

APA: Aldosterone-producing adenoma
ARR: Aldosterone renin activity ratio
AUC: Area under the ROC curve
AVS: Adrenal vein sampling
BP: Blood pressure
CCT: Captopril challenge test
CT: Computed tomography
CVs: Coefficients of variation
DBP: Diastolic blood pressure
FST: Fludrocortisone suppression test
IHA: Idiopathic hyperaldosteronism
LI: Lateralization index
NC: Normal controls
PA: Primary aldosteronism
PAC: Plasma aldosterone concentration
PH: Primary hypertension
PRA: Plasma renin activity
RAS: Renin-angiotensin system
ROC: Receiver-operated characteristic
SBP: Systolic blood pressure
SI: Selectivity index
SIT: Saline infusion test
